# Radiotherapy delays malignant transformation and prolongs survival in patients with IDH-mutant gliomas

**DOI:** 10.20892/j.issn.2095-3941.2022.0472

**Published:** 2022-11-01

**Authors:** Yanwei Liu, Huiyuan Chen, Guanzhang Li, Jing Zhang, Kun Yao, Chenxing Wu, Shouwei Li, Xiaoguang Qiu

**Affiliations:** 1 Department of Radiation Oncology, Beijing Tiantan Hospital, Capital Medical University, Beijing 100070, China; 2 National Clinical Research Center for Neurological Diseases, Beijing 100070, China; 3 Department of Neuropathology, Beijing Neurosurgical Institute, Capital Medical University, Beijing 100070, China; 4 Department of Neurosurgery, Beijing Tiantan Hospital, Capital Medical University, Beijing 100070, China; 5 Department of Neuropathology, Sanbo Brain Hospital, Capital Medical University, Beijing 100093, China; 6 Department of Neurosurgery, Sanbo Brain Hospital, Capital Medical University, Beijing 100093, China

**Keywords:** Lower-grade gliomas, *IDH* mutation, radiotherapy, malignant transformation, survival

## Abstract

**Objective:**

IDH-mutant lower-grade gliomas (LGGs, grade 2 or 3) eventually transform into secondary grade 4 astrocytomas (sA_IDHmut/G4_). Here, we sought to describe the transformation time, risk factors, and outcomes in malignant transformation of IDH-mutant LGGs.

**Methods:**

We screened data for 108 patients with sA_IDHmut/G4_ in the Chinese Glioma Genome Atlas who had initial IDH-mutant LGGs and underwent reoperation during 2005–2021. We evaluated the transformation time from IDH-mutant LGGs to sA_IDHmut/G4_, and associated risk factors and outcomes. Malignant transformation was defined as pathological confirmation of grade 4 astrocytoma.

**Results:**

The median age of the 108 patients with IDH-mutant LGGs was 35 years (range, 19–54); the median age at transformation was 40 years (range, 25–62); and the median follow-up time for all patients was 146 months (range, 121–171). The average transformation time was 58.8 months for all patients with LGGs (range, 5.9–208.1); 63.5 and 51.9 months for grade 2 and 3 gliomas, respectively; and 58.4 and 78.1 months for IDH-mutant/1p/19q-non-codeleted astrocytomas and IDH-mutant/1p/19q-codeleted oligodendrogliomas, respectively. Univariate and multivariate analysis indicated that radiotherapy [hazard ratio (HR), 0.29; 95% confidence interval (CI), 0.137–0.595; *P* = 0.001] and non-A blood type (HR, 0.37; 95% CI, 0.203–0.680; *P* = 0.001) were protective factors against delayed malignant transformation. Radiotherapy was associated with improved survival after transformation (HR, 0.44; 95% CI, 0.241–0.803; *P* = 0.008), overall survival (HR, 0.50; 95% CI, 0.265–0.972; *P* = 0.041), and progression-free survival (HR, 0.25; 95% CI, 0.133–0.479; *P* < 0.0001) in patients with IDH-mutant gliomas.

**Conclusions:**

Radiotherapy is associated with delayed malignant transformation and improved survival in patients with IDH-mutant gliomas.

## Introduction

The *isocitrate dehydrogenase 1/2* (*IDH*) mutation is the main distinguishing feature of lower-grade gliomas (LGGs, grade 2 or 3 astrocytomas/oligodendrogliomas) and secondary glioblastomas that developed from LGGs [according to the 2016 World Health Organization (WHO) classification]^1^. According to the 2021 WHO classification for tumors of the central nervous system, grading of diffuse gliomas relies not only on histological appearance but also on genetic parameters^2^. *IDH* mutation is a critical factor in diagnosis, which is used to guide glioma treatment and clinical trial eligibility. IDH-mutant LGGs undergo malignant transformation and eventually progress to secondary grade 4 IDH-mutant astrocytoma (sA_IDHmut/G4_)^3^. However, the term “secondary glioblastoma” is no longer applied to central nervous system WHO grade 4 IDH-mutant astrocytoma with histological evidence of preceding LGGs, and has not been discussed in the current classification. The characteristics for malignant transformation from IDH-mutant LGGs to sA_IDHmut/G4_ have not been systematically studied.

In this study, we sought to systematically elucidate the clinical process of malignant transformation from LGGs to sA_IDHmut/G4_. To our knowledge, the present study is the largest paired cohort study of IDH-mutant LGG and sA_IDHmut/G4_ to date. We evaluated the transformation time, risk factors, and outcomes. Our findings may provide a reference illustrating malignant transformation and aid in development of therapeutic strategies for IDH-mutant gliomas.

## Materials and methods

### Patients

For this multicenter retrospective analysis, we collected 108 pairs of IDH-mutant LGG and sA_IDHmut/G4_ from the Chinese Glioma Genome Atlas, which was initiated by the Chinese Glioma Cooperative Group during 2005–2021 (www.cgga.org.cn/). Histopathology was confirmed independently by 2 neuropathologists (Huiyuan Chen and Kun Yao) on the basis of the 2021 WHO classification. Malignant transformation was defined as pathological confirmation of grade 4 astrocytoma. We defined the following inclusion criteria: (1) age > 18 years and presence of supratentorial gliomas; (2) diagnosis based on surgical specimens (excluding biopsy specimens); (3) sA_IDHmut/G4_ diagnosed through both microvascular proliferation and necrosis, and a history of LGGs; (4) testing of tumor tissue for *IDH* mutation (*IDH1* and *IDH2*) by pyrosequencing. Three-dimensional conformal radiotherapy or intensity-modulated radiation therapy was conducted at a dose of 50.4–60.0 Gy (1.8–2.0 Gy daily, 5 days per week). A total of 37.0% of the patients received chemotherapy at the time of initial diagnosis, whereas 75.0% of the patients received chemotherapy after transformation to sA_IDHmut/G4_ (**Table 1**). Carmustine, nimustine, or temozolomide was used as chemotherapy. The study protocol was approved by the Ethics Review Board of Beijing Tiantan Hospital in Beijing, China. Written informed consent was obtained from all participants. The patients were required to be in good general condition with Karnofsky Performance Score ≥ 60.

**Table 1:** Characteristics of 108 patients with LGGs

Characteristics	*n* (%)
Median age at initial diagnosis (LGGs)	35 (19–54)
Median age at transformation (sA_IDHmut/G4_)	40 (25–62)
Gender	
Male	68 (63.0%)
Female	40 (37.0%)
Blood type	
A	27 (25.0%)
B	26 (24.1%)
AB	14 (13.0%)
O	31 (28.7%)
NA	10 (9.3%)
Location 1	
Left	48 (44.4%)
Right	57 (52.8%)
Both	3 (2.8%)
Location 2	
Single lobe	77 (71.3%)
Across multiple lobes	31 (28.7%)
Grade	
Grade 2	74 (68.5%)
Grade 3	22 (20.4%)
Grade 2 or 3	12 (11.1%)
Histological description*	
A/AA	73 (67.6%)
O/AO	4 (3.7%)
OA/AOA	26 (24.1%)
NA	5 (4.6%)
Chemotherapy at initial diagnosis (LGGs)	
Yes	40 (37.0%)
No	55 (50.9%)
NA	13 (12.0%)
Chemotherapy at transformation (sA_IDHmut/G4_)	
Yes	81 (75.0%)
No	19 (17.6%)
NA	8 (7.4%)
Radiotherapy at initial diagnosis (LGGs)	
Yes	78 (72.2%)
No	24 (22.2%)
NA	6 (5.6%)
Radiotherapy at transformation (sA_IDHmut/G4_)	
Yes	18 (16.7%)
No	90 (83.3%)
1p/19q-codeleted	
Yes	15 (13.9%)
No	79 (73.1%)
NA	14 (13.0%)
MGMT promoter methylation	
Yes	65 (60.2%)
No	25 (23.1%)
NA	18 (16.7%)

*A, astrocytoma; AA, anaplastic astrocytoma; O, oligodendroglioma; AO, anaplastic oligodendroglioma; OA, oligoastrocytoma; AOA, anaplastic oligoastrocytoma; NA, not applicable.

### Molecular detection

(1) DNA pyrosequencing for *IDH1/2* mutations: Genomic DNA was isolated from frozen tumor tissues with a QIAamp DNA Mini Kit (Qiagen). The genomic region spanning wild-type R132 of IDH1 and R172 of IDH2 was amplified with the following primers: IDH1-F-5′-GCTTGTGAGTGGATGGGTAAAAC-3′ and R-5′-TTGCCAACATGACTTACTTGATC-3′; and IDH2-F-5′-ATCCTGGGGGGGACTGTCT T-3′ and R-5′-CTCTCCACCCTGGCCTACCT-3′. Single-stranded DNA was purified from the total PCR products and subjected to pyrosequencing on a PyroMark Q96 ID System (QIAGEN) with the primers IDH1-5′-TGGATGGGTAAAACCT-3′ and IDH2-5′-AGCCCATCACCATTG-3′.

(2) Fluorescence *in situ* hybridization for 1p/19q codeletion: Sections (4 μm) cut from formalin-fixed and paraffin-embedded (FFPE) tissue blocks were analyzed through fluorescence *in situ* hybridization with locus specific identifier probe sets 1p36/1q25 and 19q13/19p13 (spectrum orange-labeled 1p36 and 19q13 probes; spectrum green-labeled 1q25 and 19p13 probes; Vysis), and at least 200 non-overlapping nuclei with intact morphology were evaluated. A deletion ratio of > 30% was considered positive.

(3) Pyrosequencing of the MGMT promoter: DNA was extracted from FFPE tissue samples with a QIAamp DNA FFPE Tissue Kit (Qiagen, Hilden, Germany). Bisulfite conversion of 100 ng DNA was performed with an Epitect Bisulfite kit (Qiagen, Hilden, Germany) according to the manufacturer’s protocol, and was followed by amplification and pyrosequencing. Briefly, the bisulfite-treated DNA was amplified with the forward primer 5′-GTTTYGGATATGTTGGGATAGTT-3′ and the biotinylated reverse primer 5′-biotin-ACRACCCAAACACTCACCAA-3′. Two independent assays were performed in different samples with 2 pyrosequencing sequencing primers: 5′-GATATGTTGGGATAG T-3′ (for CpGs 72–78) and 5′-GTTTTTAGAAYGTTTTG3′ (for CpGs 75–82). The methylation levels of CpG sites 76–79 were detected with a commercial MGMT pyrosequencing kit (#970061, Qiagen, Hilden, Germany) and a PyroMark Q24 System (Qiagen, Hilden, Germany). A cutoff of ≥ 10% was used to define a “methylated” MGMT promoter. MGMT methylation was detected in 30 sA_IDHmut/G4_ samples through immunohistochemistry (MGMT mouse monoclonal antibody, #ZM-0461, ZSGB-BIO). The staining intensity was jointly scored by 2 neuropathologists (Huiyuan Chen and Kun Yao) on a scale of -, +, ++, and +++ (-/+, methylation; ++/+++, non-methylation) (**Supplementary Figure S1**).

### Statistical analysis

Overall survival (OS), progression-free survival (PFS), and survival after transformation were analyzed with the Kaplan–Meier method, and groups were compared with a log-rank test. OS was calculated from the date of surgery to the date of death. PFS was calculated from the day of surgery to the first event. Transformation time was calculated from the initial diagnosis of the LGGs to the diagnosis of sA_IDHmut/G4_. Cox proportional hazards regression was used to identify independent risk factors. Statistical significance was set at *P* < 0.05 (two-sided).

## Results

### Patient characteristics

Patient characteristics are presented in **Table 1**. The median follow-up time for all patients was 146 months (range, 121 –171). The median age at initial diagnosis of LGGs and transformation to sA_IDHmut/G4_ was 35 years (range, 19–54) and 40 years (range, 25–62), respectively. The male-to-female ratio was 1.7:1 (68:40), and 74 (68.5%) patients had WHO grade 2, 22 (20.4%) patients had grade 3, and 12 patients had grade 2 or 3 (which could not be further specified because of limited information) LGGs. Of the 108 IDH-mutant LGGs, 15 (13.9%) had a 1p/19q codeletion, and 79 (73.1%) had no codeletion. According to histopathological classification, 99 patients (91.7%) were diagnosed with astrocytoma or mixed astrocytoma, 3 with grade 2 oligodendroglioma and one with grade 3 oligodendroglioma.

### Malignant transformation

**Figure 1:**
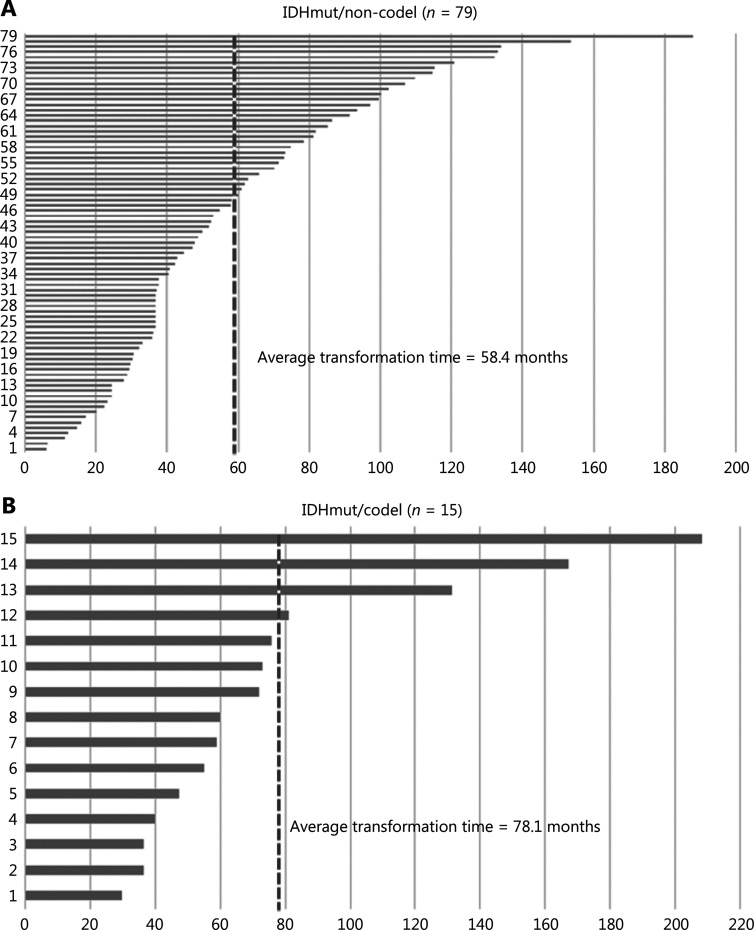

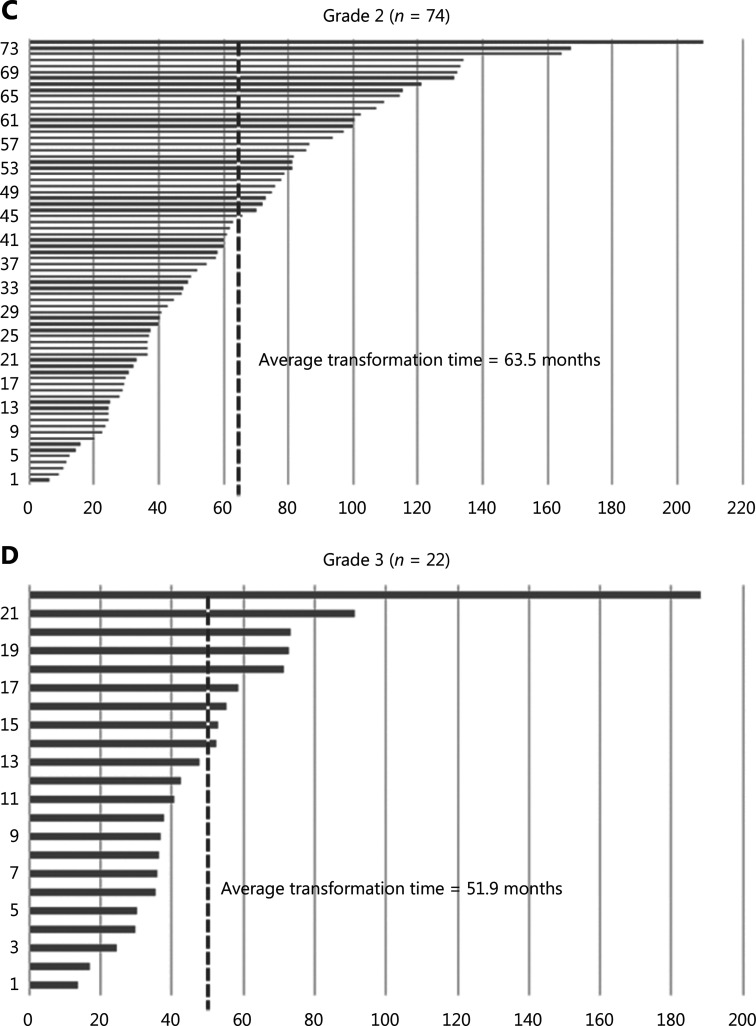
Malignant transformation from LGGs to sA_IDHmut/G4_. Transformation time from the 1p/19q non-codeleted or codeleted LGGs to sA_IDHmut/G4_ (A-B), and from grade 2 gliomas and 3 gliomas to sA_IDHmut/G4_ (C-D). LGGs, lower-grade gliomas.

**Figure 2:**
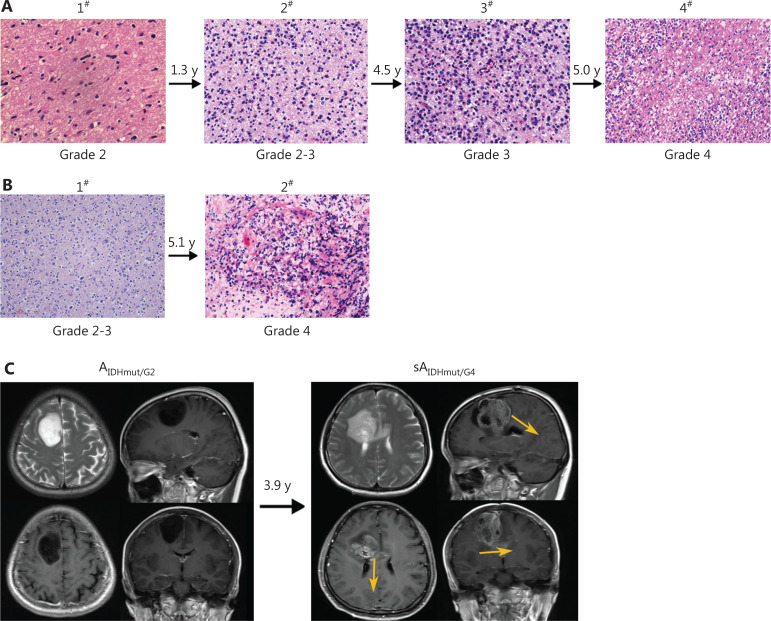
Histological and MRI analysis of malignant transformation. (A, B) Histological analysis of tumor samples from 2 patients who underwent 4 and 2 surgical resections from grade 2 or 3 glioma to sA_IDHmut/G4_ (200×). (C) MRI scan showing progressive characteristics in LGG and sA_IDHmut/G4_. ^#^operations. MRI, magnetic resonance imaging; LGG, lower-grade glioma.

The average time from diagnosis to transformation was 58.8 months for all patients with IDH-mutant LGGs (range, 5.9–208.1); 58.4 and 78.1 months for 1p/19q-non-codeleted and codeleted LGGs, respectively (**Figure 1A and 1B**); and 63.5 and 51.9 months for grade 2 and grade 3 gliomas, respectively (**Figure 1C and 1D**). The transformation time did not significantly differ between grade 2 and grade 3 gliomas (**Supplementary Figure S2A**; HR, 0.68; *P* = 0.163), or between 1p/19q-non-codeleted and codeleted LGGs (**Supplementary Figure S2B**; HR, 0.67; *P* = 0.121). However, patients with IDH-mutant/codeleted oligodendroglioma had longer survival times after transformation than patients with IDH-mutant/non-codeleted astrocytoma (**Supplementary Figure S2C**; 28.3 months *vs*. 10.8 months; HR, 0.67; *P* = 0.041); the PFS after transformation was also longer in IDH-mutant/codeleted oligodendroglioma (**Supplementary Figure S2D**; *P* = 0.068). Among the 108 patients, a total of 139 reoperations were performed (**Figure 2A and 2B**), and 14 patients underwent multiple reoperations (> 2). Magnetic resonance imaging (MRI) indicated that LGGs tended to extend into adjacent lobes or into the opposite hemisphere through the cingulate gyrus or the corpus callosum, thus indicating progression (**Figure 2C**).

### Radiotherapy delays malignant transformation and improves survival

**Figure 3:**
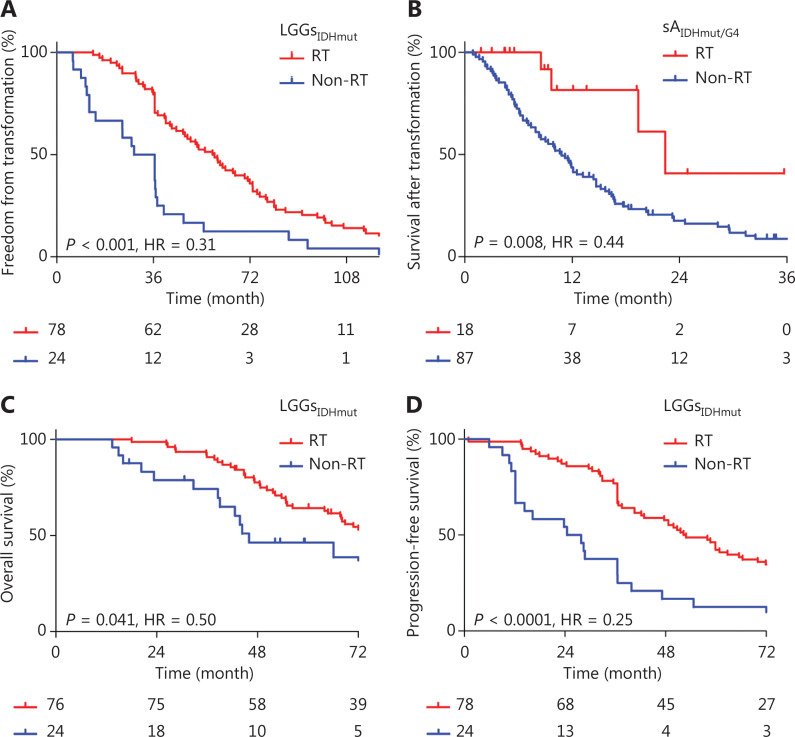
Radiotherapy delays malignant transformation and improves survival in IDH-mutant gliomas. Radiotherapy is associated with delayed malignant transformation (A), improved survival after transformation (B), improved overall survival (C), and progression-free survival (D).

Univariate and multivariate analysis based on the age, sex, grade, left *vs.* right location, single *vs*. multiple lobe location, blood type (B/O *vs.* A/AB), chemotherapy, radiotherapy, 1p/19q codeletion, and MGMT promoter methylation showed that radiotherapy (HR, 0.29; 95% CI, 0.137–0.595; *P* = 0.001) and non-A blood type (HR, 0.37; 95% CI, 0.203–0.680; *P* = 0.001) were independent factors associated with delayed transformation (**Table 2**). Kaplan–Meier analysis indicated that the time for transformation was 58.4 months in patients with LGG treated with radiotherapy compared with 32.6 months in patients without radiotherapy (HR, 0.31; 95% CI, 0.165–0.565; *P* < 0.001) (**Figure 3A**). Radiotherapy was also associated with improved survival after transformation in 18 patients who received reirradiation when tumors transformed into sA_IDHmut/G4_ (HR, 0.44; 95% CI, 0.241–0.803; *P* = 0.008) (**Figure 3B**); the PFS after transformation was also longer in patients with reirradiation (**Supplementary Figure S3A**; HR, 0.50; *P* = 0.014). In addition, for all patients with IDH-mutant LGGs, radiotherapy was associated with both improved OS (**Figure 3C**; HR, 0.50; *P* = 0.041) and PFS (the first recurrence) (**Figure 3D**; HR, 0.25; *P* < 0.0001).

**Table 2:** Univariate and multivariate analyses for malignant transformation based on clinical and molecular variables

Variables		Univariate			Multivariate		
*n*/108	HR	95% CI	*P*	HR	95% CI	*P*
Age (19–54, years)	108/108	1.011	0.989–1.033	0.348	1.000	0.962–1.040	0.997
Gender							
Male *vs*. female	108/108	1.161	0.781–1.725	0.461	1.347	0.740–2.453	0.330
Grade	96/108						
3 *vs*. 2	74 *vs*. 22	1.420	0.879–2.295	0.152	1.645	0.877–3.089	0.121
Location 1	108/108						
Left *vs*. right	45 *vs*. 63	1.099	0.750–1.611	0.627	1.300	0.703–2.405	0.403
Location 2	107/108						
Single *vs*. multiple lobe	75 *vs*. 32	0.721	0.473–1.099	0.128	0.552	0.229–1.019	0.058
Radiotherapy	102/108						
Yes *vs*. no	78 *vs*. 24	0.499	0.313–0.795	0.003	0.285	0.137–0.595	0.001
Chemotherapy	97/108						
Yes *vs*. no	41 *vs*. 56	1.093	0.726–1.645	0.670	1.540	0.860–2.757	0.146
Blood type	95/108						
B/O *vs*. A/AB	39 *vs*. 56	0.589	0.385–0.903	0.015	0.372	0.203–0.680	0.001
MGMT promoter methylation	90/108						
Yes *vs*. no	65 *vs*. 25	0.979	0.663–1.446	0.914	0.596	0.653–2.101	0.596
1p/19q co-deletion	94/108						
Yes *vs*. no	15 *vs*.79	0.648	0.363–1.155	0.141	0.788	0.387–1.603	0.511

## Discussion

To our knowledge, this study is the largest analysis of malignant transformation based on paired IDH-mutant LGG and sA_IDHmut/G4_ cohorts. We illustrated the clinical process of malignant transformation from LGG to sA_IDHmut/G4_. This is the first study reporting the transformation time and the association of radiotherapy with delayed malignant transformation and improved survival in patients with IDH-mutant gliomas.

Patients with LGGs with *IDH* mutation show favorable survival^1^, but survival is substantially shortened when LGGs transform into sA_IDHmut/G4_^4–6^. Patients with grade 2 or 3 gliomas received radiotherapy or chemotherapy alone at initial diagnosis, and most patients received chemotherapy alone when their LGGs transformed to sA_IDHmut/G4_ (**Table 1**). Patients with sA_IDHmut/G4_ are more resistant to chemoradiotherapy because of patients’ treatment history^7^. Therefore, delayed malignant transformation is the main concern in improving survival for patients with LGGs. *IDH* mutation is a definitive molecular marker of gliomas. Therapies specifically targeting this mutant molecule might achieve antitumor activity. Preclinical studies have shown that inhibitors of mutant *IDH* delay the growth of IDH-mutant glioma cells^8^. However, testing of these inhibitors is ongoing in phase I/II studies^9^. Recently, treatment of glioma with an IDH1-specific peptide vaccine has achieved 63% 3-year PFS and 84% 3-year OS among patients^10^. IDH inhibitors and vaccines are expected to guide patient selection in the future. In addition, retrospective and prospective trials have shown that patients with IDH-mutant grade 2 and 3 gliomas have better responses to chemoradiotherapy than those with wild-type gliomas^11,12^. However, how to inhibit malignant transformation of IDH-mutant gliomas has not been reported, although several findings have indicated molecular mechanisms^4,13^. Yu et al.^6^ have reported a median time of 5.9 years from diagnosis to transformation (grade 3 or 4 gliomas), in 66 grade 2 gliomas. Martin et al.^14,15^ have reported 5-year freedom from malignant transformation (grade 3 or 4 gliomas) of 86%, in 84 grade 2 gliomas. However, the identification of recurrence or transformation in these studies was based on either stereotactic biopsy or increased contrast enhancement on MRI. Distinguishing radionecrosis from tumor progression through MRI is difficult^16,17^; furthermore, the histopathology of sA_IDHmut/G4_ is highly variable, thus hindering diagnosis based on stereotactic needle biopsy specimens^18,19^. In our study, the grading and classification were confirmed through histopathological diagnosis with specimens obtained from craniotomy (gross total or subtotal resection). We identified 108 patients with initial grade 2 or 3 IDH-mutant gliomas who underwent at least 2 operations because of progression or transformation. The time from diagnosis to transformation is longer in patients with than without prior radiotherapy. Reirradiation was also associated with improved survival after transformation in IDH-mutant LGGs.

Previous studies before the era of molecular classification showed that radiotherapy significantly improves PFS but not OS in patients with grade 2 gliomas^20,21^. Molecular-based classification has been established in gliomas, particularly LGGs, and with the use of molecular biomarkers, gliomas can be differentiated more accurately in terms of their prognosis and response to different therapies^4^. In our study, the association of radiotherapy with improved OS and PFS was observed in patients with IDH-mutant LGGs. Patients with an *IDH* mutation/1p/19q non-codeletion or wild-type *IDH* may have improved survival with the administration of high-dose radiotherapy^22,23^. Moreover, MGMT status is an independent prognostic biomarker of LGGs treated with radiotherapy^24^. Therefore, molecular classification plays an important role in predicting radiotherapy effects, and new trials should be designed in the current molecular era. In addition, interestingly, the transformation time from IDH-mutant LGG to sA_IDHmut/G4_ was longer in patients with blood type non-A (O/B) than patients with blood type A/AB (57.8 months *vs*. 37.6 months; HR, 0.44; *P* = 0.011) (**Supplementary Figure S3B**). ABO blood type is associated with various diseases, including cardiovascular disease, tumors, and immune diseases. Several studies have shown an association of blood group A with a higher risk of gastric cancer^25^. Moreover, the ABO blood group has been associated with pancreatic cancer, nasopharyngeal carcinoma, and ovarian and lung cancers^26^. However, the role of the ABO blood group in gliomas has not yet been described in relevant reports.

We acknowledge the limitations of our retrospective cohort design, including our inability to retest 1p/19q codeletion in all sA_IDHmut/G4_ samples. However, *IDH* mutations are stable in both primary and progressive/recurrent gliomas^27^. Oligodendrogliomas are molecularly defined by the presence of *IDH* mutation and 1p/19q codeletion. No classification exists for grade 4 oligodendroglioma because of its favorable prognosis, despite pathological microvascular proliferation and necrosis. However, 15 LGGs had *IDH* mutation and 1p/19q codeletion, and eventually transformed into sA_IDHmut/G4_ in our study. This apparent contradiction occurred more frequently before the molecular classification era^28,29^, and these 1p/19q codeletion-defined oligodendrogliomas probably contained an unrecognized astrocytoma component. Glioblastomas with an oligodendroglioma component are frequently observed in clinical practice^30^. Whether the characteristic of 1p/19q codeletion might be lost during malignant transformation must be studied further.

## Conclusions

We illustrated the clinical process of malignant transformation in patients with IDH-mutant LGGs and reported the transformation time. Importantly, we highlighted the effects of radiotherapy in delaying transformation and improving survival in patients with IDH-mutant gliomas. Our findings help clarify the clinical process of malignant transformation and may aid in the development of therapeutic strategies for IDH-mutant gliomas.
