# Supplementary material for: Radiotherapy delays malignant transformation and prolongs survival in patients with IDH-mutant gliomas
Source: Cancer Biol Med. 2022 Nov 1;19(10):1477–86. doi: 10.20892/j.issn.2095-3941.2022.0472 (PMC9630524; doi:10.20892/j.issn.2095-3941.2022.0472)
Supplement: Supplementary file 1 [file cbm-19-1477-s001.pdf]

Supplementary materials

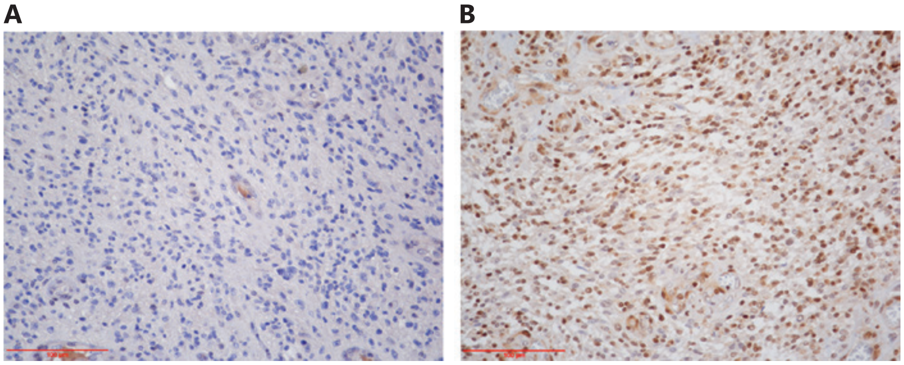

Figure S1 MGMT promoter methylation, validated by immunohistochemistry. Negative and methylation (A); positive and non-methylation (B).

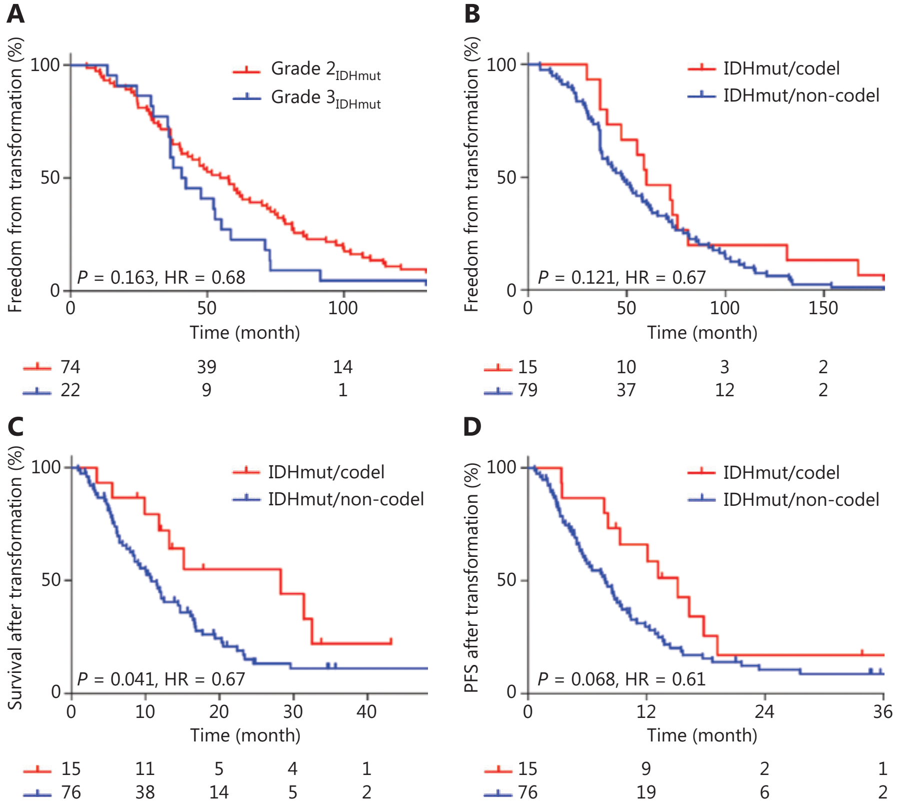

Figure S2 Malignant transformation from LGGs to $sA_{IDHmut/G4}$. Kaplan-Meier estimates of transformation time between grade 2 and 3 gliomas (A) and between 1p/19q non-codeleted LGGs and codeleted LGGs (B). Kaplan-Meier estimates of overall survival (C) and progression-free survival (D) after transformation between 1p/19q non-codeleted LGGs and codeleted LGGs.

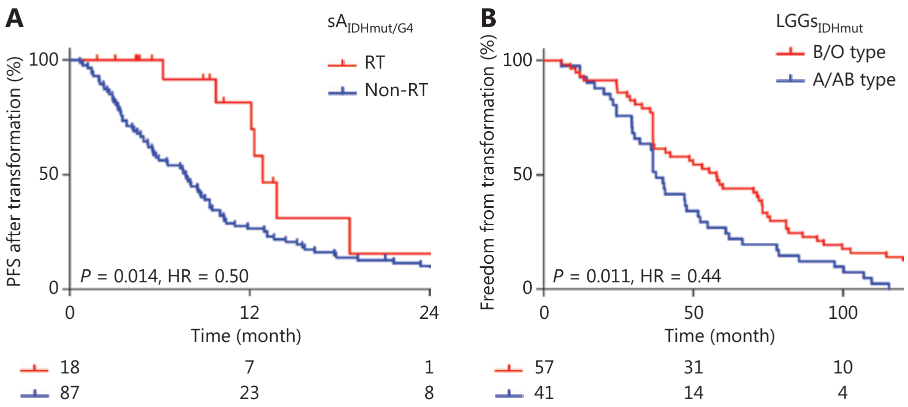

Figure S3 Survival analysis based on different subgroups. (A) Radiotherapy was associated with improved progression-free survival after transformation. (B) Transformation time was shorter in patients with blood type A/AB than in patients with blood type O/B.
